# A nested case-control study of the effects of dust exposure, smoking on COPD in coal workers

**DOI:** 10.1186/s12889-023-16944-6

**Published:** 2023-10-20

**Authors:** Hui Wang, Rui Meng, Xuelin Wang, Zhikang Si, Zekun Zhao, Haipeng Lu, Huan Wang, Jiaqi Hu, Yizhan Zheng, Jiaqi Chen, Ziqi Zhao, Hongmin Zhu, Xiaoming Li, Ling Xue, Shengguang Yan, Jian Sun, Yu Su, Jianhui Wu

**Affiliations:** 1 School of Public Health, Caofeidian New Town, North China University of Science and Technology, No.21 Bohai Avenue, Tangshan City, Hebei Province 063210 People’s Republic of China; 2 Personnel Department, North China University of Science and Technology, Tangshan City, Hebei Province, China; 3 School of Public Health, North China University of Science and Technology, Tangshan, Hebei Province China

**Keywords:** Coal workers, Dust exposure, Smoking, COPD, Interaction

## Abstract

**Background:**

Chronic obstructive pulmonary disease (COPD) represents a prevalent ailment, progressively surging within the ranks of coal mine laborers. The current study endeavors to elucidate the effects of dust exposure and smoking on COPD incidence amongst coal mine workers, while concurrently devising preventive strategies for this affliction.

**Method:**

A nested case–control study was conducted encompassing 1,416 participants aged ≥ 18 years, spanning the duration from (2017–2018) until 2020. A meticulous matching process yielded a cohort of 708 COPD patients, each paired with a control subject, forming a harmonious 1:1 ratio. Multiple logistic regression analysis was employed to scrutinize the associations between smoking, dust exposure with COPD among coal workers.

**Results:**

The COPD prevalence within the cohort of coal workers under investigation amounted to 22.66%, with an accompanying incidence density of 0.09/person-year. Following meticulous adjustment for confounding variables, it was discerned that cumulative dust exposure within the range of 47.19 ~ (OR: 1.90, 95% CI: 1.05, 3.44), 101.27 ~ (OR: 1.99, 95% CI: 1.17, 3.39), as well as smoking indices of 72 ~ (OR: 1.85, 95% CI: 1.19, 2.88), 145 ~ (OR: 1.74, 95% CI: 1.17, 2.61), 310 ~ (OR: 1.85, 95% CI: 1.23, 2.77) engender an escalated vulnerability to COPD among coal workers. Furthermore, interaction analysis discerned an absence of both multiplicative and additive interactions between dust exposure, smoking, and COPD occurrence amidst coal workers.

**Conclusion:**

Dust exposure and smoking were unequivocally identified as precipitating risk factors for COPD incidence within the population of coal workers, albeit devoid of any discernible interaction between these two causal agents.

**Supplementary Information:**

The online version contains supplementary material available at 10.1186/s12889-023-16944-6.

## Background

Chronic obstructive pulmonary disease (COPD) represents a pervasive global ailment and stands as a prominent contributor to the burden of disease worldwide, owing to its high mortality and disability rates. It is characterized by persistent limitation of airflow and accompanied by symptoms including dyspepsia, chronic cough, and expectoration. In 2004, a survey reported a global prevalence of COPD in adults aged ≥ 40 years to be approximately 9% ~ 10% [1]. Subsequent investigations have indicated a progressive escalation in COPD prevalence, reaching 13.1% in 2019 [2]. A comprehensive study encompassing seven regions in China unveiled an overall COPD prevalence of 13.6% among individuals aged 40 years or older in 2015, with the southwest region exhibiting an alarmingly high prevalence of 20.2% [3]. Moreover, COPD emerged as a leading cause of disease burden in China, accounting for 934,000 deaths and 16,723,800 disability adjusted life years (DALYs) in 2010 [4]. Financially, the annual per capital direct medical expenses for COPD patients range from $72 ~ $3565, accounting a substantial portion of the local per capita annual income, ranging from 33.33% to 118.09% [5]. It is important to note that COPD not only impairs lung function but also gives rise to numerous complications, including cardiovascular disease, lung cancer, osteoporosis and depression [6]. These compelling data warrant vigilant attention to the management and prevention of COPD.

Smoking has long been recognized as a prominent risk factor for chronic diseases, including Cardiovascular Disease [7], renal injury [8], digestive system diseases [9], cancer [10], and COPD [11]. A nested case–control study in a Chinese occupational population indicated that more tobacco smoked per day, per year, and over a lifetime meant a higher chance of developing COPD, with an OR of 4.60 for those with > 40 pack-years of smoking who also had the highest metal exposures. Currently, due to the high incidence rate of COPD, more and more scholars are committed to finding new risk factors, particularly within occupational settings [12, 13]. According to a study, occupational exposure accounts for 15% ~ 20% of COPD cases, with mineral dust exposure alone amplifying the risk by 72% [14, 15]. Considering the significance of coal as a vital global energy source, responsible for 27.62% of worldwide energy supply and fulfilling 60% of China's energy demands [16]. It is crucial to acknowledge the sizable population of coal workers in China. These workers, due to their unique occupational environment, encounter heightened exposure to mineral dust and chemical toxins, thereby increasing their susceptibility to pneumococcal infections and COPD. In fact, a cross-sectional investigation conducted within the Chinese population revealed a staggering prevalence of 32.7% for the coexistence of COPD and pneumoconiosis among coal miners [17].

Currently, a considerable proportion of individuals suffering from COPD lack awareness of the condition and fail to undergo a pulmonary function test prior to its onset. Furthermore, their access to relevant medical resources remains limited. Remarkably, a study reported that merely 2.4% of COPD patients have ever undergone a pulmonary function test, and a mere 7.9% of patients with GOLD stage II or higher regularly receive appropriate medication [18]. Despite the insidious nature of COPD and its challenging identification, it is important to recognize that this disease is preventable through behavioral and lifestyle modifications. Therefore, the exploration of COPD risk factors assumes paramount significance in promoting health and enhancing the quality of life for coal workers. Currently, most studies have employed a cross-sectional design to investigate COPD risk factors. However, this design is insufficient to fully elucidate the causal relationship between risk factors and outcomes.

Hence, the present study endeavors to employ a nested case–control design to explore the impact of dust exposure and smoking on COPD among coal mine workers. By doing so, it aims to provide actionable strategies for COPD prevention among coal workers, reducing the incidence of this condition, and optimizing the utilization of healthcare resources.

## Materials and methods

### Study participants

Exploring the impact of dust exposure and smoking on COPD among coal mine workers is of great significance for promoting the health of coal workers. Therefore, the nested case–control was conducted by the following methods. A nested case–control study was carried out using data from an ongoing prospective cohort study with 3501 dust-exposed workers aged 18 + years in a coal mine situated in Xingtai City, Hebei Province, China. The inclusion criteria encompassed workers aged between 18 and 60 years, with a minimum of one year of service. Conversely, individuals who were unable to undergo a spirometry test due to recent chest, abdominal, or eye surgeries within the preceding three months, those who were pregnant or breastfeeding, and those who had been hospitalized for heart disease within the previous month were excluded from the study. Furthermore, individuals who lacked essential information as per the questionnaires were also excluded from the cohort.

**Fig. 1:**
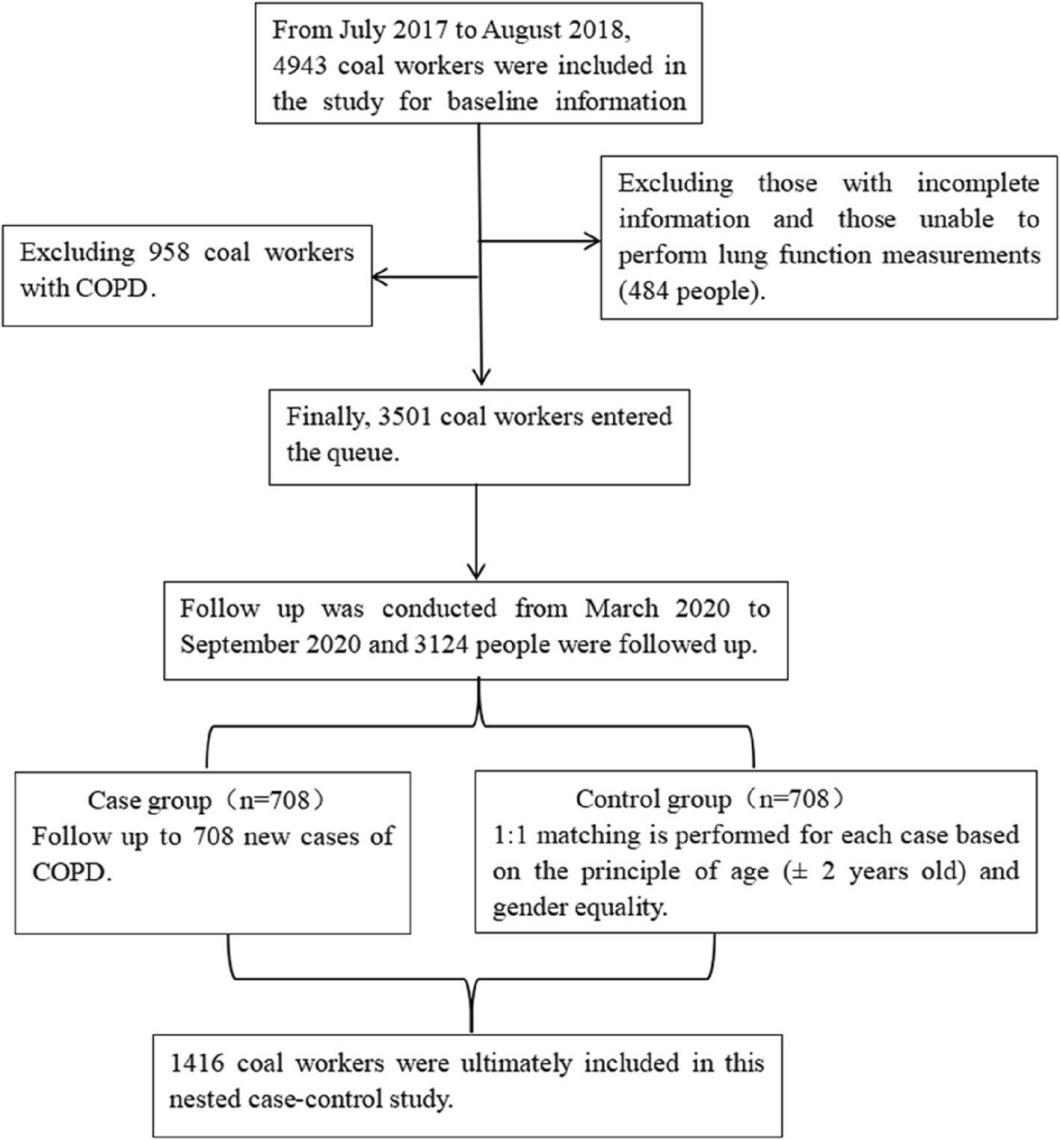
Study design and selection process of the study participants

The survey was conducted to follow up on the cohort from July 2017 to August 2018, extending until March 2020 to September 2020. Out of the original 3,501 participants, 3,124 individuals were successfully followed, resulting in a loss rate of 10.77%. Among these followed individuals, the case group comprised 708 newly diagnosed COPD patients during the follow-up period, while the control group consisted of 708 coal workers who remained free of COPD. The case group was meticulously matched with the control group in a 1:1 ratio, based on the principle of identical sex and age (± 2 years). Ultimately, a total of 1,416 individuals were included in this nested case–control study (Fig. 1). Prior to participation, all individuals provided informed consent. The study was conducted in strict adherence to the ethical guidelines outlined in the Declaration of Helsinki and was approved by the Medical Ethics Committee of the North China University of Technology.

### Information collection

The data collection process encompassed various components, including questionnaire survey, physical examinations, laboratory tests, and on-site hygiene surveys. The questionnaire survey comprised several key aspects: (1) Demographic information: This section covered details such as age, gender, ethnicity, marital status, educational attainment, and economic income. (2) Participants were queried about their smoking status, alcohol consumption, dietary habits, physical activity levels, and sleep patterns. (3) Personal and family disease history: Information regarding personal medical history as well as family histories of conditions such as hypertension, diabetes, tumors, and other diseases were recorded. (4) Work situation: Participants were asked about the nature of their employment, length of service, type of work, and their shift schedules. The physical examination involved varioust measurement, including height, weight, blood pressure, and pulmonary function assessments. Laboratory examinations encompassed blood routine tests, urine routine tests and blood biochemical analyses. The on-site hygiene survey primarily focused on evaluating the working environment of coal workers. This entailed assessing levels of dust, noise, and exposure to high temperatures. By employing these comprehensive data collection methods, a holistic understanding of the participants' characteristics, behaviors, health statuses, and work conditions could be obtained.

Pulmonary function tests were conducted utilizing a portable respirometer known as CHEST. This device measured two key parameters the first second expiatory volume (FEV_1_) and the forceful lung capacity (FVC). Prior to administering the pulmonary function tests, trained professionals provided a comprehensive explanation of the necessary precautions and demonstrated the required movements to the subjects. To ensure the accuracy of the instrument, simulation tests were performed by the professionals before initiating the actual testing. During the test, the subjects maintained an upright position, with their upper body kept still in a quiet seated posture. Following the commands of the staff, the subjects cooperated by inhaling as deeply as possible and exhaling forcefully and rapidly through a mouthpiece, while their nose was temporarily clamped. The staff members operated the respirometer throughout the procedure, and upon completion of the test, the results were printed out for further analysis.

### Exposure assessment

(1) Cumulative dust exposure (CDE): The criteria delineated in the "Determination of Dust in Workplace Air, Part 1: Total Dust Concentration". To calculate an individual's cumulative exposure to dust, a qualified testing company measures the total dust concentration in the workplace through daily actual testing [19]. The standard for dust concentration is determined based on the measurements conducted by the qualified testing company. In order to establish quartile groups, the CDE values are divided into the following categories: 0, 0.1 ~ , 18.07 ~ , 47.19 ~ , 101.27 ~ . To ensure a comprehensive representation of the distribution, the upper quartile is divided by the median value. As a result, the CDE values are categorized as follows: 0, 0.1 ~ , 18.07 ~ , 47.19 ~ , 101.27 ~ . This grouping strategy enables the inclusion of the tail distribution of the data.

1 $$\mathrm{CDE}={\mathrm{C}}_{1}*{\mathrm{T}}_{1}+{\mathrm{C}}_{2}*{\mathrm{T}}_{2}+{\mathrm{C}}_{3}*{\mathrm{T}}_{3}+...+{\mathrm{C}}_{\mathrm{n}}{\mathrm{T}}_{\mathrm{n}}$$

C_n_ is the annual geometric mean concentration in mg/m^3^ for a job performed by a coal worker; T_n_ is the duration of dust pick-up in years for a job performed by a worker, in years.

Smoking Index (SI): Smoking Index = Daily Smoking Index * Number of years smoked. When utilizing the quartile method for grouping, the smoking index is stratified into the following categories: 0, 1 ~ , 72 ~ , 145 ~ , 310 ~ . To ensure a comprehensive representation of the data distribution, the upper quartile is divided by the median value, resulting in the smoking index groups of 0, 1 ~ , 39 ~ , 72 ~ , 145 ~ , 310 ~ .

(3) Smoking status: Smoking status classified as never smoking, ever smoking, and current smoking. "Never smoking" signifies a complete absence of smoking history. "Ever smoking" pertains to individuals who have engaged in smoking in the past but have since ceased this habit. "Current smoking" indicates active smoking at the time of the survey, encompassing a minimum consumption of one cigarette per day for a duration of six months or longer.

(4) Alcohol drinking status: Alcohol drinking status is categorized as never drinking, ever drinking, and currently drinking. "Never drinking" signifies a complete absence of alcohol consumption. "Ever drinking" refers to individuals who have consumed alcohol in the past but have abstained for a minimum of six months at the time of the survey. "Currently drinking" denotes the consumption of alcohol at the time of the survey, with a frequency of at least once a week on a consistent basis.

(5) High temperature exposure: As defined in this study, the determination of high temperature exposure refers to the “Workplace Physical Factor Measurement Part 7: High Temperature”. [20]. Specifically, high temperature exposure is characterized by engaging in work involving a significant heat source, whereby the WBGT reaches or exceeds 25 ℃.

(6) Noise exposure: The criteria for determining noise exposure in this study are based on the. “Measurement of physical factors in the workplace Part 8: Noise”. [21] Noise exposure is characterized by the presence of detrimental sound levels that pose a risk to hearing within the work environment. Specifically, it refers to working in an environment where the equivalent sound level is equal to or exceeds 80 dB(A) for a duration of 40 h per week or 8 h per day.

### Outcome

According to the Global Initiative for Chronic Obstructive Lung Disease (GOLD) guidelines [22], FEV_1_ /FVC < 70% was defined as COPD.

Furthermore, the GOLD guidelines are also used to categorize the severity of airflow obstruction in patients with COPD. The staging criteria are as follows: GOLD stage I: FEV_1_ ≥ 80% FEV_1_ predicted, GOLD stage II: 50% FEV_1_ predicted ≤ FEV_1_ < 80% FEV_1_ predicted, GOLD stage III: 30% FEV_1_ predicted ≤ FEV_1_ < 50% FEV_1_ predicted, GOLD stage IV: FEV_1_ < 30% FEV_1_ predicted.

### Statistical analysis

Quantitative variables were presented using medians and interquartiles range (IQR). Categorical variables were described using Frequency and percentage (%). For comparisons between groups regarding quantitative variables, the Wilcoxon rank sum test was employed. Chi-square tests were used for comparisons between groups regarding categorical variables. Multifactor analysis was conducted using conditional logistic regression, with a significance level (α) set at 0.05. The interaction between variables was assessed through the cross-product term in the conditional logistic regression. The assessment of additive interaction involved calculating the relative excess risk of interaction (RERI), attributable proportion of interaction (AP), and synergy index (S), using Andersson's self-developed EXCEL additive interaction formula.

To comprehensively understand the impact of dust exposure and smoking on COPD among coal workers, this study quantified dust exposure and smoking as cumulative dust exposure and smoking index, respectively. The relationship between dust exposure, smoking index, and COPD was examined from various perspectives, considering both quantitative and categorical variables. When categorical variables were used, dust exposure and smoking were categorized into quartiles as non-exposure and exposure. To capture the tail distribution effectively, the upper quartile was further divided by its median, resulting in a total of six categories. All statistical analyses were performed by IBM SPSS 22.0 and Excel 2019. *P* < 0.05 were considered statistically significant.

## Results

### General characteristics

The average duration of followed-up for coal workers were 2.5 years, and a total of 1416 participants were included in the cohort. Among them, there were 708 new cases of COPD, resulting in a prevalence rate of 22.66% and an incidence density of 0.091/person-year. Table 1 provided an overview of the general demographic characteristics of the study participants. The proportion of rural residents was higher in the case group (38.0%) compared to the control group (31.2%), and this difference was statistically significant (*P* = 0.009). Regarding monthly household income, the proportion of individuals earning 8000 ~ was 36.7%, which was higher compared to the control group. In terms of education level, the case group had a higher proportion (55.4%) of individuals with education beyond high school, compared to the control group (42.9%). Moreover, the proportion of individuals with a personal history of respiratory disease was higher in the case group (60.3%) compared to the control group (54.9%). These differences in residential address, monthly household income, education level, and personal history of respiratory disease between the case and control groups were statistically significant (*P* < 0.05).

**Table 1:** General demographic characteristics of the case and control groups [n(%)]

Variables	Total	Case group ( *n* = 708)	Control group ( *n* = 708)	^*2*^	*P*
Residential address				7.190	0.009
Urban	926 (65.4)	439 (62.0)	487 (68.8)		
Rural	490 (34.6)	269 (38.0)	221 (31.2)		
Marital status					
Unmarried	64 (4.5)	38 (5.4)	26 (3.7)	3.360	0.186
Married	1293 (91.3)	637 (90.0)	656 (92.7)		
Other	59 (4.2)	33 (4.7)	26 (3.7)		
Monthly household income				18.314	< 0.001
< 5000	146 (10.3)	92 (13.0)	54 (7.6)		
5000 ~	327 (23.1)	168 (23.7)	159 (22.5)		
6000 ~	483 (34.1)	248 (35.0)	235 (33.2)		
8000 ~	460 (32.5)	200 (28.2)	260 (36.7)		
Education level					
< High School	696 (49.2)	392 (55.4)	304 (42.9)	35.244	< 0.001
High School	384 (27.1)	193 (27.3)	191 (27.0)		
> High School	336 (23.7)	123 (17.4)	213 (30.1)		
Hypertension				1.479	0.224
No	1268 (89.5)	627 (88.6)	641 (90.5)		
Yes	148 (10.5)	81 (11.4)	67 (9.5)		
Diabetes				2.202	0.138
No	966 (68.2)	470 (66.4)	496 (70.1)		
Yes	450 (31.8)	238 (33.6)	212 (29.9)		
Personal history of respiratory disease				4.176	0.041
No	600 (42.4)	281 (39.7)	319 (45.1)		
Yes	816 (57.6)	427 (60.3)	389 (54.9)		
BMI				5.160	0.160
Low weight	44 (3.1)	27 (3.8)	17 (2.4)		
Normal weight	576 (40.7)	277 (39.1)	299 (42.2)		
Overweight	566 (40.0)	296 (41.8)	270 (38.1)		
Obesity	230 (16.2)	108 (15.3)	122 (17.2)		

### Analysis of differences in smoking, dust situation

Table 2 presented a comparison of dust exposure and smoking between the case and control groups from various perspectives. In terms of smoking status, the proportion of current smokers was 57.6% in the case group and 45.1% in the control group. The median smoking index in the case group (60) was significantly higher than in the control group (28), indicating a statistically significant difference (*P* = 0.017). When categorizing the smoking index into six categories, the proportion of individuals with a smoking index of 310 ~ was 16.0% in the case group, higher than the 13.1% observed in the control group. However, there was not much difference in the proportion of individuals with a smoking index of 39 ~ between the two groups. The smoking index demonstrated a statistically significant difference between the case and control groups (*P* < 0.001). Regarding cumulative dust exposure, the median and IQR among all participants were was 47.19 (18.07, 101.27) mg/m^3^. years. The median cumulative dust exposure in the case group (50.82 mg/m^3^. years) was higher than that in the control group (44.02 mg/m^3^. years). In the case group, the proportions of individuals exposed to cumulative dust levels of 47.19 ~ and 101.27 ~ were 24.2% and 30.1%, respectively, which were higher than the proportions in the control group (21.2% and 20.2%). These differences were statistically significant (*P* < 0.001).

**Table 2:** Comparison of different dust exposure and smoking indicators between the case and control groups [n(%)]

Variables	Total	Case group (*n* = 708)	Control group (*n* = 708)	^*2*^*/Z*	*P*
Smoking status				28.604	< 0.001
Never	545 (38.5)	224 (31.6)	321 (45.3)		
Ever	144 (10.2)	76 (10.7)	68 (9.6)		
Current	727 (51.3)	408 (57.6)	319 (45.1)		
Smoking Index	40 (0, 188.25)	60 (0, 210.00)	28 (0, 159.00)	-2.391	0.017
Smoking Index^a^					
0	545 (38.5)	224 (31.6)	321 (45.3)	34.729	< 0.001
1 ~	304 (21.5)	154 (21.8)	150 (21.2)		
72 ~	163 (11.5)	94 (13.3)	69 (9.7)		
145 ~	198 (14.0)	123 (17.4)	75 (10.6)		
310 ~	206 (14.5)	113 (16.0)	93 (13.1)		
Smoking Index^b^				34.879	< 0.001
0	545 (38.5)	224 (31.6)	321 (45.3)		
1 ~	127 (9.0)	66 (9.3)	61 (8.6)		
39 ~	177 (12.5)	88 (12.4)	89 (12.6)		
72 ~	163 (11.5)	94 (13.3)	69 (9.7)		
145 ~	198 (14.0)	123 (17.4)	75 (10.6)		
310 ~	206 (14.5)	113 (16.0)	93 (13.1)		
Cumulative dust exposure (mg/m^3^. years)	47.19 (18.07, 101.27)	50.82 (22.24, 101.27)	44.02 (16.68, 101.20)	-2.296	0.022
Cumulative dust exposure^a^(mg/m^3^. years)				31.383	< 0.001
0	153 (10.8)	57 (8.1)	96 (13.6)		
0.1 ~	290 (20.5)	140 (19.8)	150 (21.2)		
18.07 ~	296 (20.9)	127 (17.9)	169 (23.9)		
47.19 ~	321 (22.7)	171 (24.2)	150 (21.2)		
101.27 ~	356 (25.1)	213 (30.1)	143 (20.2)		
Cumulative dust exposure^b^				31.415	< 0.001
0	153 (10.8)	57 (8.1)	96 (13.6)		
0.1 ~	130 (3.5)	62 (8.8)	68 (9.6)		
7.79 ~	160 (11.3)	78 (11.0)	82 (11.6)		
18.07 ~	296 (20.9)	127 (17.9)	169 (23.9)		
47.19 ~	321 (22.7)	171 (24.2)	150 (21.2)		
101.27 ~	356 (25.1)	213 (30.1)	143 (20.2)		

^a^Converting cumulative dust exposure into categorical variable, i.e., non-exposure and quartile of exposure; Converting the smoking index into categorical variable, i.e. non-smoking and quartile of smoking

^b^On the basis of the a classification method, the upper quartile of exposure to cumulative dust exposure and smoking index was divided by the median into two categories

## Analysis of the association between dust exposure, smoking and COPD

**Fig. 2:**
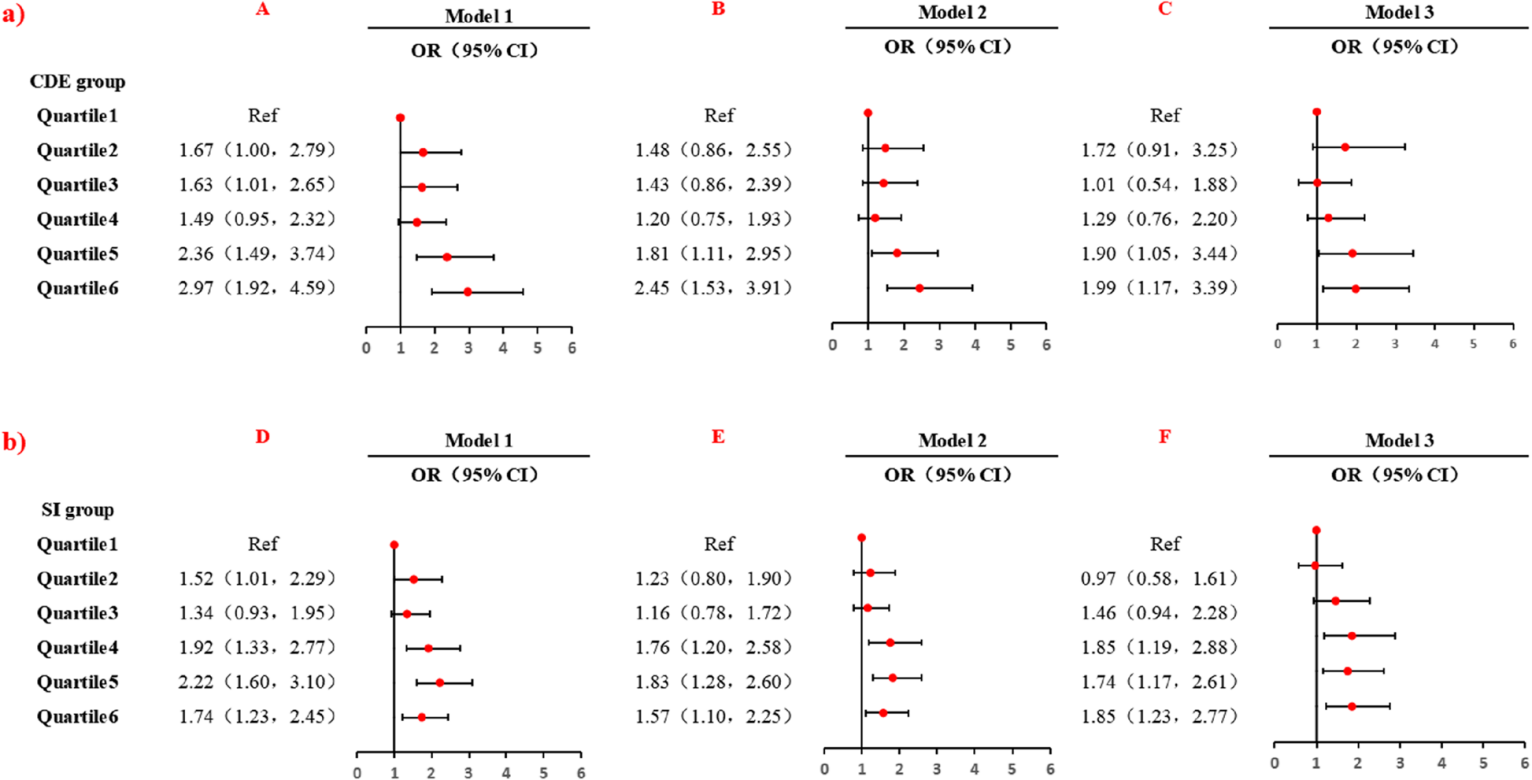
Analysis of the association between cumulative dust exposure and smoking index and COPD among coal workers. Model 1: crude model; Model 2: adjusted for residential address, education level, monthly household income, and personal history of respiratory disease; Model 3: further adjusted for alcohol drinking status, smoking index or cumulative dust exposure, frequency of vegetable consumption, frequency of fruit consumption, physical exercise, chemical toxicant exposure, seniority, physical activity, mask usage, and ventilation and dust removal measures. **a**) The association between cumulative dust exposure and COPD among coal workers. **b**) The association between smoking index and COPD among coal workers

Unadjusted and adjusted multivariate logistic regression models were employed for analysis (Figure 2). The unadjusted Model 1 demonstrated a significant association between COPD and cumulative dust exposure levels of 7.79~ (OR = 1.63, 95% CI: 1.01 - 2.65), 47.19~ (OR = 2.36, 95% CI: 1.49 - 3.74) and 101.27~ (OR = 2.97, 95% CI: 1.92 - 4.59). However, after adjusting for covariates, cumulative dust exposure level of 7.79~ was no longer associated with an increased risk of COPD. Cumulative dust exposure levels of 7.19~ and 101.27~ consistently exhibited significant roles in the risk of COPD among coal workers across all models. Model 3 revealed an increased hazard for coal workers exposed to cumulative dust exposure levels of 47.19~ (OR = 1.90, 95% CI: 1.05 - 3.44) and 101.27~ (OR = 1.99, 95% CI: 1.17 - 3.39). Smoking indices of 72~, 145~, and 310~ were associated with an elevated risk of COPD in coal workers. In the fully adjusted Model 3, the odds ratios for smoking indices of 72~, 145~ and 310~ were 1.85 (95%: 1.19 - 2.88), 1.74 (95%: 1.17 - 2.61) and 1.85 (95%: 1.23 - 2.77), respectively.

In Table 3, we observed a relationship between cumulative dust exposure, smoking index, and the severity of airflow restriction within the case group. Notably, there was a statistically significant increase in the risk of airflow restriction severity associated with cumulative dust exposure. The smoking index also exhibited an association with the risk of airflow restriction severity. The proportion of coal workers with cumulative dust exposure of 101.27~ in GOLD stage III group (45.4%) was the highest among the three groups, with a statistically significant difference (*P* < 0.001). Additionally, the proportion of coal workers with a smoking index 310~ in GOLD stage I and II groups was similar but significantly lower than the proportion in the GOLD stage III group (20.5%, *P* = 0.015). The detailed process of univariate and multivariate regression analysis is presented in the supplementary materials (Table S 1 - Table S 3).

**Table 3:** Analysis of the association between cumulative dust exposure, smoking and COPD severity in the case group [n(%)]

Variables	GOLD stage I	GOLD stage II	GOLD stage III	^*2*^	*P*
	(*n* = 235)	(*n* = 288)	(*n* = 185)		
Cumulative dust exposure (mg/m^3^. years)				32.543	< 0.001
0	22 (9.4)	25 (8.7)	10 (5.4)		
0.1 ~	24 (10.2)	26 (9.0)	12 (6.5)		
7.79 ~	24 (10.2)	32 (11.1)	22 (11.9)		
18.07 ~	46 (19.6)	55 (19.1)	26 (14.1)		
47.19 ~	58 (24.7)	82 (28.5)	31 (16.8)		
101.27 ~	61 (26.0)	68 (23.6)	84 (45.4)		
Smoking Index				21.994	0.015
0	88 (37.4)	91 (31.6)	45 (24.3)		
1 ~	22 (9.4)	32 (11.1)	17 (9.2)		
39 ~	31 (13.2)	38 (13.2)	15 (8.1)		
72 ~	27 (11.5)	43 (14.9)	24 (13.0)		
145 ~	34 (14.5)	43 (14.9)	46 (24.9)		
310 ~	33 (14.0)	41 (14.2)	38 (20.5)		

The 2017 Global Initiative for Chronic Obstructive Lung Disease (GOLD) guidelines was used to stage the degree of obstruction. GOLD stage I: FEV_1_≧80% predicted; GOLD stage II: 50% predicted≦FEV_1_ < 80% predicted; GOLD stage III: due to the small number of GOLD stage IV, GOLD stage IV and GOLD stage III were merged into GOLD stage III, FEV_1_ < 50% predicted was defined as GOLD stage III

### Interaction analysis of smoking and dust exposure

Multiplicative interactions were analyzed using fully adjusted conditional logistic regression models, incorporating the cross product term (Table 4). However, no significant multiplicative interaction was observed between cumulative dust exposure, smoking index, and COPD (*P*_multiplication_ = 0.181 > 0.05). As for additive interactions, it is important to note that the additive interaction model can only assess interactions between dichotomous variables. Therefore, we combined the variables "ever smoking" and "current smoking" into a smoking group. Furthermore, all three measures of interaction (RERI [-0.08, -1.58, 1.42], AP [-0.02, -0.38, 0.34], and S [0.98, 0.62, 1.54]) indicated no evidence of additive interaction (Table 5).

**Table 4:** Analysis of the multiplicative interaction between cumulative dust exposure and smoking index on COPD in coal workers

Cumulative dust exposure	Smoking Index	OR (95% CI)	*P*_multiplication_
			0.181
0	0	1.00	
0.1 ~	1 ~	0.53 (0.12, 2.90)	
0.1 ~	39 ~	1.09 (0.15, 7.80)	
0.1 ~	72 ~	1.95 (0.33, 11.54)	
0.1 ~	145 ~	0.32 (0.03, 2.95)	
0.1 ~	310 ~	0.58 (0.07, 4.69)	
7.79 ~	1 ~	0.50 (0.08, 3.33)	
7.79 ~	39 ~	0.28 (0.03, 2.43)	
7.79 ~	72 ~	0.60 (0.08, 4.72)	
7.79 ~	145 ~	0.20 (0.03, 1.47)	
7.79 ~	310 ~	0.13 (0.02, 1.03)	
18.07 ~	1 ~	0.17 (0.02, 1.34)	
18.07 ~	39 ~	1.83 (0.15, 22.91)	
18.07 ~	72 ~	0.49 (0.06, 4.34)	
18.07 ~	145 ~	0.43 (0.04, 5.35)	
18.07 ~	310 ~	0.54 (0.12, 2.32)	
47.19 ~	1 ~	0.95 (0.30, 3.00)	
47.19 ~	39 ~	0.40 (0.07, 2.25)	
47.19 ~	72 ~	0.53 (0.12, 2.34)	
47.19 ~	145 ~	0.32 (0.07, 1.50)	
47.19 ~	310 ~	0.86 (0.22, 3.36)	
101.27 ~	1 ~	0.50 (0.18, 1.40)	
101.27 ~	39 ~	0.15 (0.03, 0.83)	
101.27 ~	72 ~	1.27 (0.26, 6.19)	
101.27 ~	145 ~	0.23 (0.05, 1.03)	
101.27 ~	310 ~	0.21 (0.06, 0.72)	

Adjusted for residential address, education level, monthly household income, personal history of respiratory disease, frequency of vegetable consumption, frequency of fruit consumption, physical exercise, alcohol drinking status, mask usage, seniority, ventilation and dust removal measures, and chemical toxicant exposure

**Table 5:** Analysis of the additive interaction between dust exposure and smoking on COPD in coal workers

Indicators	Estimated value	(95% CI) value
RERI	-0.08	(-1.58, 1.42)
AP (%)	-0.02	(-0.38, 0.34)
S	0.98	(0.62, 1.54)

Dust exposure is divided into exposed and unexposed, with "never smoked" defined as non-smoking and "ever smoked" and "currently smoking" defined as " Smoking"

## Discussion

In order to gain a comprehensive understanding of the respective roles of dust exposure and smoking in relation to COPD among coal workers, a nested case–control study was conducted, including 708 individuals who were newly diagnosed with COPD, serving as cases, as well as 708 control subjects. The results of this study reaffirmed the association between dust exposure, smoking, and an elevated risk of COPD among coal workers. However, our analysis did not yield any evidence of either additive or multiplicative interaction between dust exposure, smoking, and the occurrence of COPD in this particular population of coal workers.

In our study, we observed a COPD incidence rate of 22.66% among coal workers, which is higher than the reported prevalence of 12% among the general Chinese population in 2019 [23]. Smoking is widely recognized as an established and primary risk factor for COPD [24–26]. The number of cigarettes smoked is positively correlated with an increased risk of COPD. Our study found that smoking index of 72 ~ , 145 ~ , and 310 ~ played significant roles in the risk for COPD among coal workers, which ialigns with previous findings. A large-scale study conducted in China demonstrated a positive assocation between smoking pack-years and COPD prevalence [27]. Similarly, the Burden Of Obstructive Lung Disease study also reported that a strong positive association between 10 pack-years and GOLD stage II (OR: 1.28, for women, OR: 1.16 for men) [28]. This can be attributed to the release of inflammatory cytokines from respiratory cells stimulated by cigarette smoke, leading to respiratory damage [29–31]. Regarding cumulative dust exposure, our study identified exposure levels of 47.19 ~ and 101.27 ~ as risk factors for COPD among coal workers. These findings are consistent with previous research indicating that dust exposure is associated with an increased risk of COPD [32, 33]. Given that coal mining is a prominent industry where workers are exposed to mineral dust, the relationship between occupation and COPD risk was investigated in the Biobank cohort study in the UK, which demonstrated an association between coal mine operatives and increased COPD risk (PR = 2.30; 95% CI: 1.00—5.31) [34]. Moreover, our study revealed that for 1 mg/m^3^.year increase in cumulative dust exposure among coal workers, the risk of COPD death increases by 1.0065 (95% CI: 1.0017—1.0054), and the risk of chronic respiratory obstruction increases by 1.0081 (95% CI: 1.0025—1.0318) [35]. Additionally, a prospective cohort study demonstrated a significant linear relationship between the duration of coal mine dust exposure and the decline in FEV_1._ [36]. The mechanism underlying the increased risk of COPD among coal miners may be attributed to exposure to coal mine dust and silica dust. Coal mine dust can inactivate alpha-1 antitrypsin, which increases the risk of COPD, and it also generates reactive oxygen species that contribute to emphysema [37]. Silica particles can induce airway inflammation and emphysema by increasing the production of oxidants, cytokines, chemokines, and elastase. Furthermore, silica particles can cause epithelial cell damage, facilitate the penetration of silica particles into small airway walls, and lead to local fibrosis [38]. Furthermore, our study observed strong associations between cumulative dust exposure, smoking index, and the severity of COPD. These findings align with a survey conducted in the Swiss cohort study, which demonstrated an association between occupational exposure to mineral dust and the incidence and severity of COPD [39].

In our study, we also investigated the potential interaction between dust exposure and smoking in relation to COPD. However, no evidence of additive or multiplicative interaction was found between smoking and dust exposure. This finding is consistent with some previous studies [39, 40]. It is possible that the absence of additive and multiplicative interaction in our study can be attributed to the fact that workers in the high dust exposure group exhibited healthier lifestyle habits. Nevertheless, conflicting results have been reported by other researchers, and some scholars have suggested that there is indeed an interaction between these two factors and COPD [41, 42]. Further research is warranted to gain a deeper understanding of the specific mechanisms involved. In an 11-year follow-up cohort study, an additive effect on the incidence of COPD was observed, with the annual incidence rate of COPD among occupationally exposed smokers being 9.03/1000 higher than 1.88/1000 among non-exposed non-smokers, which was 1.88/1000 [42]. Additionally, a study conducted on workers in Southern Italy demonstrated an interaction between smoking and occupational exposure, as evidenced by a higher prevalence of workers exposed to both risk factors [43].

One of the important strengths of our study is the utilization of a nested case–control study design to investigate the risk factors of COPD among coal workers. This design offers advantages over a cross-sectional design, as it helps to mitigate recall bias and selection bias, thereby ensuring the validity and reliability of our study findings. Another strength of our study lies in the comprehensive analysis of the effects of dust exposure and smoking on COPD among coal workers from various detailed perspectives. However, our study does have several limitations that should be acknowledged. Firstly, the lack of available data on biomass use and PM_2.5_ exposure during the follow-up period is a significant limitation. These factors have been shown to have a substantial association with an increased prevalence of COPD. Secondly, since the cases were matched with controls based on age and gender, our study did not account for the potential effects of age and gender on the prevalence of COPD.

In conclusion, our survey highlights the high prevalence of COPD among coal workers. Smoking and dust exposure are identified as significant modifiable risk factors for COPD. It is crucial to implement targeted prevention strategies among coal workers to effectively reduce the morbidity associated with COPD.

### Supplementary Information

**Additional file 1. **

## Data Availability

Data can be obtained upon a reasonable inquiry. The datasets produced and examined during the present study are not accessible to the public at this time, as further analyses are still in progress. However, they can be obtained from the corresponding author upon a reasonable request.

## Abbreviations

COPD: Chronic obstructive pulmonary disease
BMI: Body Mass Index
OR: Odds ratio
95% CI: 95% Confident limit
RERI: Relative excess risk of interaction
AP: Attributable proportion of interaction
SI: Smoking index
