# Supplementary material for: A nested case-control study of the effects of dust exposure, smoking on COPD in coal workers
Source: BMC Public Health. 2023 Oct 20;23:2056. doi: 10.1186/s12889-023-16944-6 (PMC10588135; doi:10.1186/s12889-023-16944-6)
Supplement: Supplementary file 1 — Additional file 1. [file 12889_2023_16944_MOESM1_ESM.docx]

**Table S 1** Results of behavioral lifestyle comparison between the case and control groups [n(%)]

| Variables | Total | Case group  （ n = 708） | Control group  （n = 708） | χ^2^ | P |
| --- | --- | --- | --- | --- | --- |
| Alcohol drinking status |  |  |  | 6.465 | 0.039 |
| Never | 340 (24.0) | 182 (25.7) | 158 (22.3) |  |  |
| Ever | 69 (4.9) | 42 (5.9) | 27 (3.8) |  |  |
| Current | 1007 (71.1) | 484 (68.4) | 523 (73.9) |  |  |
| Physical exercise |  |  |  | 23.351 | <0.001 |
| No | 997 (70.4) | 540 (76.3) | 457 (64.5) |  |  |
| Yes | 419 (29.6) | 168 (23.7) | 251 (35.5) |  |  |
| Sleep quality |  |  |  | 1.942 | 0.386 |
| Accessibility | 965 (68.1) | 494 (69.8) | 471 (66.5) |  |  |
| Suspicious Insomnia | 307 (21.7) | 148 (20.9) | 159 (22.5) |  |  |
| Insomnia | 144 (10.2) | 66 (9.3) | 78 (11.0) |  |  |
| Frequency of vegetable consumption |  |  |  | 38.643 | <0.001 |
| Never | 74 (5.2) | 62 (8.8) | 12 (1.7) |  |  |
| Occasionally | 95 (6.7) | 52 (7.3) | 43 (6.1) |  |  |
| Frequently | 141 (10.0) | 61 (8.6) | 80 (11.3) |  |  |
| Daily | 1106 (78.1) | 553 (75.3) | 573 (80.9) |  |  |
| Frequency of fruit consumption |  |  |  | 32.146 | <0.001 |
| Never | 187 (13.2) | 121 (17.1) | 66 (9.3) |  |  |
| Occasionally | 476 (33.6) | 250 (35.3) | 226 (31.9) |  |  |
| Frequently | 245 (16.5) | 126 (17.8) | 119 (16.8) |  |  |
| Daily | 508 (35.9) | 211 (29.8) | 297 (41.9) |  |  |
| Frequency of meat consumption |  |  |  | 8.990 | 0.029 |
| Never | 184 (13.0) | 110 (15.5) | 74 (10.5) |  |  |
| Occasionally | 636 (44.9) | 317 (44.8) | 319 (45.1) |  |  |
| Frequently | 305 (21.5) | 144 (20.3) | 161 (22.7) |  |  |
| Daily | 291 (20.6) | 137 (19.4) | 154 (21.8) |  |  |
| Frequency of consumption of soy products |  |  |  | 1.883 | 0.597 |
| Never | 231 (16.3) | 125 (17.7) | 106 (15.0) |  |  |
| Occasionally | 655 (46.3) | 323 (45.6) | 332 (46.9) |  |  |
| Frequently | 266 (18.8) | 131 (18.5) | 135 (19.1) |  |  |
| Daily | 264 (18.6) | 129 (18.2) | 135 (19.1) |  |  |

**Table S 2** Exposure to occupationally relevant factors for coal workers [n(%)]

| Variables | Total | Case group  （ n = 708） | Control group  （n = 708） | χ^2^ | P |
| --- | --- | --- | --- | --- | --- |
| Seniority(years) |  |  |  | 10.830 | 0.013 |
| <9 | 317 (22.4) | 144 (20.3) | 173 (24.4) |  |  |
| 9~ | 331 (23.4) | 150 (21.2) | 181 (25.6) |  |  |
| 13~ | 379 (26.8) | 199 (28.1) | 180 (25.4) |  |  |
| 22~ | 389 (27.5) | 215 (30.4) | 174 (24.6) |  |  |
| Ventilation and dust removal measures |  |  |  | 19.713 | <0.001 |
| difference | 113 (8.0) | 77 (10.9) | 36 (5.1) |  |  |
| General | 750 (53.0) | 380 (53.7) | 370 (52.3) |  |  |
| Good | 553 (39.1) | 251 (35.5) | 302 (42.7) |  |  |
| Mask usage |  |  |  | 19.005 | <0.001 |
| Never | 264 (18.6) | 157 (22.2) | 107 (14.9) |  |  |
| 1-2 days/week | 152 (10.7) | 84 (11.9) | 68 (9.6) |  |  |
| 3-4/week | 60 (4.2) | 21 (3.0) | 39 (5.5) |  |  |
| Daily | 940 (66.4) | 446 (63.0) | 494 (69.8) |  |  |
| chemical poison exposure |  |  |  | 4.159 | 0.041 |
| No | 806 (56.9) | 384 (54.2) | 422 (59.6) |  |  |
| Yes | 610 (43.1) | 324 (45.8) | 286 (40.4) |  |  |
| Noise exposure |  |  |  | 0.941 | 0.332 |
| No | 592 (41.8) | 287 (40.5) | 305 (43.1) |  |  |
| Yes | 824 (58.2) | 421 (59.5) | 403 (56.9) |  |  |
| High temperature exposure |  |  |  | 0.794 | 0.373 |
| No | 496 (35.0) | 256 (36.2) | 240 (33.9) |  |  |
| Yes | 920 (65.0) | 452 (63.8) | 468 (66.1) |  |  |
| Shift situation |  |  |  | 1.692 | 0.429 |
| Never | 411 (29.0) | 205 (29.0) | 206 (29.1) |  |  |
| Once | 115 (8.1) | 51 (7.2) | 64 (9.0) |  |  |
| Now | 890 (62.9) | 452 (63.8) | 438 (61.9) |  |  |
| Physical activity |  |  |  | 9.461 | 0.009 |
| Low | 222 (15.6) | 124(17.5) | 98(13.8) |  |  |
| Middle | 127(9.0) | 75(10.6) | 52 (7.3) |  |  |
| High | 1067 (75.4) | 509 (71.9) | 558 (78.8) |  |  |

**Table S 3** Results of multi-factorial conditional logistic regression analysis of COPD in coal workers

| Variables | β | SE_β_ | Waldχ^2^ | *P* | OR (95% CI) |
| --- | --- | --- | --- | --- | --- |
| Residential address |  |  |  |  |  |
| Urban | - | - | - | - | 1.00 |
| Rural | 0.318 | 0.157 | 5.890 | 0.015 | 1.46  (1.07, 1.99) |
| Education level |  |  |  |  |  |
| < High School | - | - | - | - | 1.00 |
| High School | -0.282 | 0.162 | 3.041 | 0.081 | 0.75  (0.55, 1.04) |
| > High School | -0.543 | 0.203 | 7.017 | 0.008 | 0.59  (0.40, 0.87) |
| Monthly household  income |  |  |  |  |  |
| <5000 | - | - | - | - | 1.00 |
| 5000~ | -0.354 | 0.248 | 2.050 | 0.152 | 0.70  (0.43, 1.14) |
| 6000~ | -0.321 | 0.238 | 1.820 | 0.177 | 0.73  (0.46, 1.16) |
| 8000~ | -0.501 | 0.234 | 4.577 | 0.032 | 0.61  (0.38, 0.96) |
| Personal history of respiratory disease |  |  |  |  |  |
| No | - | - | - | - | 1.00 |
| Yes | 0.467 | 0.142 | 10.872 | 0.001 | 1.60  (1.21, 2.11) |
| Alcohol drinking status |  |  |  |  |  |
| Never | - | - | - | - | 1.00 |
| Ever | 0.388 | 0.334 | 1.346 | 0.246 | 0.68  (0.35, 1.31) |
| Current | 0.413 | 0.171 | 5.833 | 0.016 | 0.66  (0.47, 0.93) |
| Frequency of vegetable consumption |  |  |  |  |  |
| Never | - | - | - | - | 1.00 |
| Occasionally | -0.632 | 0.467 | 1.829 | 0.176 | 0.53  (0.21, 1.33) |
| Frequently | -1.057 | 0.445 | 5.629 | 0.018 | 0.35  (0.15, 0.83) |
| Daily | -0.988 | 0.401 | 6.062 | 0.014 | 0.37  (0.17, 0.82) |
| Frequency of fruit consumption |  |  |  |  |  |
| Never | - | - | - | - | 1.00 |
| Occasionally | -0.467 | 0.210 | 4.945 | 0.026 | 0.63  (0.42, 0.95) |
| Frequently | -0.610 | 0.240 | 6.472 | 0.011 | 0.54  (0.34, 0.87) |
| Daily | -0.913 | 0.213 | 18.399 | <0.001 | 0.40  (0.26, 0.61) |
| Frequency of meat consumption |  |  |  |  |  |
| Never | - | - | - | - | 1.00 |
| Occasionally | -0.364 | 0.206 | 3.145 | 0.076 | 0.70  (0.46, 1.04) |
| Frequently | -0.407 | 0.233 | 3.046 | 0.081 | 0.67  (0.42, 1.05) |
| Daily | -0.448 | 0.234 | 3.668 | 0.055 | 0.64  (0.40, 1.01) |
| Physical exercise |  |  |  |  |  |
| No | - | - | - | - | 1.00 |
| Yes | -0.314 | 0.151 | 4.325 | 0.038 | 0.73  (0.54, 0.98) |
| Seniority |  |  |  |  |  |
| <9 | - | - | - | - | 1.00 |
| 9~ | -0.262 | 0.222 | 1.394 | 0.238 | 0.77  (0.500, 1.19) |
| 13~ | 0.269 | 0.267 | 1.015 | 0.314 | 1.31  (0.78, 2.21) |
| 22~ | 0.677 | 0.303 | 4.982 | 0.026 | 1.97  (1.09, 3.57) |
| Ventilation and dust removal measures |  |  |  |  |  |
| Difference | - | - | - | - | 1.00 |
| General | -0.470 | 0.275 | 2.923 | 0.087 | 0.63  (0.37, 1.08) |
| Good | -0.728 | 0.275 | 6.988 | 0.008 | 0.48  (0.28, 0.83) |
| Mask usage |  |  |  |  |  |
| Never | - | - | - | - | 1.00 |
| 1-2 days/week | -0.575 | 0.257 | 5.001 | 0.025 | 0.56  (0.34, 0.93) |
| 3-4/week | -1.069 | 0.347 | 9.522 | 0.002 | 0.34  (0.17, 0.68) |
| Daily | -0.544 | 0.184 | 8.512 | 0.004 | 0.58  (0.40, 0.84) |
| Chemical poison exposure |  |  |  |  |  |
| No | - | - | - | - | 1.00 |
| Yes | 0.720 | 0.201 | 12.764 | <0.001 | 2.05  (1.38, 3.05) |
| Cumulative dust exposure  (mg/m^3^. years) |  |  |  |  |  |
| 0 | - | - | - | - | 1.00 |
| 0.1~ | 0.532 | 0.329 | 2.609 | 0.106 | 1.70  (0.89, 3.25) |
| 7.79~ | 0.007 | 0.324 | 0.000 | 0.983 | 1.01  (0.53, 1.90) |
| 18.07~ | 0.298 | 0.273 | 1.188 | 0.276 | 1.35  (0.79, 2.30) |
| 47.19~ | 0.677 | 0.307 | 4.883 | <0.001 | 1.97  (1.08, 3.59) |
| 101.27~ | 0.732 | 0.277 | 6.999 | <0.001 | 2.08  (1.21, 3.58) |
| Smoking Index |  |  |  |  |  |
| 0 | - | - | - | - | 1.00 |
| 1~ | -0.049 | 0.260 | 0.036 | 0.850 | 0.95  (0.57, 1.58) |
| 39~ | 0.430 | 0.231 | 3.454 | 0.063 | 1.54  (0.98, 2.42) |
| 72~ | 0.604 | 0.228 | 7.025 | 0.008 | 1.83  (1.17, 2.86) |
| 145~ | 0.525 | 0.209 | 6.345 | 0.012 | 1.69  (1.12, 2.54) |
| 310~ | 0.627 | 0.210 | 8.948 | 0.003 | 1.87  (1.24, 2.82) |
| Physical activity |  |  |  |  |  |
| Low | - | - | - | - | 1.00 |
| Middle | -0.305 | 0.277 | 1.213 | 0.271 | 1.36  (0.79, 2.33) |
| High | -0.315 | 0.177 | 3.158 | 0.730 | 0.73  (0.52, 1.03) |
